# Significant expansion of the REST/NRSF cistrome in human versus mouse embryonic stem cells: potential implications for neural development

**DOI:** 10.1093/nar/gkv514

**Published:** 2015-05-18

**Authors:** Shira Rockowitz, Deyou Zheng

**Affiliations:** 1 Department of Genetics, Albert Einstein College of Medicine, Bronx, NY 10461, USA; 2 Department of Neurology, Albert Einstein College of Medicine, Bronx, NY 10461, USA; 3 Department of Neuroscience, Albert Einstein College of Medicine, Bronx, NY 10461, USA

## Abstract

Recent studies have employed cross-species comparisons of transcription factor binding, reporting significant regulatory network ‘rewiring’ between species. Here, we address how a transcriptional repressor targets and regulates neural genes differentially between human and mouse embryonic stem cells (ESCs). We find that the transcription factor, Repressor Element 1 Silencing Transcription factor (REST; also called neuron restrictive silencer factor) binds to a core group of ∼1200 syntenic genomic regions in both species, with these conserved sites highly enriched with co-factors, selective histone modifications and DNA hypomethylation. Genes with conserved REST binding are enriched with neural functions and more likely to be upregulated upon REST depletion. Interestingly, we identified twice as many REST peaks in human ESCs compared to mouse ESCs. Human REST cistrome expansion involves additional peaks in genes targeted by REST in both species and human-specific gene targets. Genes with expanded REST occupancy in humans are enriched for learning or memory functions. Analysis of neurological disorder associated genes reveals that Amyotrophic Lateral Sclerosis and oxidative stress genes are particularly enriched with human-specific REST binding. Overall, our results demonstrate that there is substantial rewiring of human and mouse REST cistromes, and that REST may have human-specific roles in brain development and functions.

## INTRODUCTION

Differential wiring of transcriptional regulatory networks and turnover of regulatory elements are hypothesized to be critical evolutionary mechanisms. Numerous studies have investigated the conservation and divergence of transcription factor (TF) targeted gene networks. Cross-species comparative analyses in metazoans using chromatin immunoprecipitation (ChIP) coupled with deep sequencing (ChIP-seq) (1–10) has shown that the conservation of transcription factor binding sites (TFBSs) between humans and mice is generally small. While sequence divergence between species strongly affects binding site conservation of tissue-specific TFBSs such as those of CEBPA and HNF4α (4), ‘new-born’ tissue-independent TFBS motifs, e.g. those of CTCF, are functionally similar to ultra-conserved ones (5). Interestingly, an analysis of six functionally diverse TFs: GATA1, SOX2, CTCF, MYC, MAX and ETS1, concluded that genes with hominid-specific binding sites were preferentially involved in neurological pathways and enriched with neural and sensory-related functions (11). This result suggests that some hominid-specific TFBSs may converge on regulating human brain development and mediating human behavior. Overall, these previous studies indicate that many TF regulatory networks have been significantly rewired during evolution. In contrast, post-translational core histone modifications (HMs) have higher conservation and co-localization across species than TFs (12). In this work, we address the issue of the evolution of gene regulation by studying a transcription factor that is critical for neurogenesis and neural homeostasis.

The Repressor Element 1 Silencing Transcription factor (REST; also known as neuron restrictive silencer factor, NRSF) binds to a 21 bp (base pair) sequence called the RE1 (Repressor Element 1) and interacts with chromatin modifiers to regulate gene expression. It plays important roles in stem cell function, cell differentiation and cancer development, but is best studied in the repression of neural genes in non-neuronal cell types (13–26). Genome-wide analyses of REST occupancy by chromatin immunoprecipitation across diverse tissues and cell types, however, have found that only a limited fraction of REST binding is targeted to the same neuronal genes in different cell types (23,24). This finding indicates that while REST has some core tissue-independent functions, it also targets and potentially regulates a wide variety of genes in a tissue-specific manner.

The transcriptional effect of REST binding on its targets is also complex and context-dependent. Although conventionally considered a repressor, in some cell types and at some specific sites REST can activate its targets (26). Moreover, even at some of the most rigorously characterized targets, REST confers different degrees of gene repression by recruiting different co-factors (27). ChIP analysis of eight REST target genes (e.g. *Bdnf, Scn2a, L1cam, Scg10*) in MHP36 murine neural stem cells identified four configurations of REST co-factors (27). Similarly, a genome-wide survey found that only the REST binding sites in mouse ESCs with the strongest binding and RE1 motifs had any repressive co-factor assembly (25). Our recent study of REST binding across 16 human cell types further demonstrated that REST interacts with different co-factors, such as SIN3 and HDACs, in a cellular and genomic context-dependent manner (24).

TF regulatory network re-wiring has previously been studied in the context of the RE1 motifs, given the known importance of REST in neural system development. Mortazavi *et al*. found that diverse genomes were similarly enriched in RE1 motifs and that a significant proportion of motifs are within repeat families (28). Johnson *et al*. investigated the dynamics of RE1 sites in humans and mice and reported that RE1 sites have experienced significant transposable element (TE) assisted expansion (29). Interestingly, some species-specific RE1 sites have undergone purifying selection and many primate-specific RE1s have emerged proximal to neural genes (30). Cross-species comparison of REST occupancy and targets have also been carried out previously, but to a very limited extent (30). As previous studies have been focused on RE1 motif conservation rather than the conservation of actual REST occupancy, it remains unclear whether REST binding events are more or less conserved than their cognate RE1 motifs. As REST is a key regulator of neuron specification and maintenance, it will be extremely important to study what genes and pathways REST specifically targets in humans and whether the REST regulatory network has experienced more rewiring than other TF networks. Furthermore, since REST functions as a hub for chromatin-modifying complexes and the regulatory outcome on its targets is largely dependent on the presence of other co-factors (26), it will be of particular interest to study how the co-localization of REST, its co-factors and chromatin modifications are conserved at REST binding sites across species. These are the key issues that we set out to investigate through a comprehensive comparison of REST-bound genomic regions in human and mouse ESCs.

## MATERIALS AND METHODS

### ChIP-seq data analysis and peak calling

We downloaded ChIP-seq reads from the Gene Expression Omnibus (31) and aligned them to the human (GRCh37/hg19) or mouse (GRCm38/mm10) genomes using Bowtie (32). Unique reads mapped to a single genomic location from the best stratum (allowing up to three mismatches) were kept for peak identification. When replicates existed, peaks were called on pooled reads from all replicates. We called peaks using the MACS algorithm (version 1.4) (33), the SICER algorithm (version 1.1) (34) or the spp pipeline (35) (modified from version 1.10 by Anshul Kundaje) with IDR guidelines (36). We used SICER for ChIP-seq data of HMs and histone deacetylases, MACS for samples with < 10 million pooled reads and spp with IDR for the rest of the ChIP-seq samples (Supplementary Table S1). The importance of IDR in determining optimal peak calling parameters has been described previously (37). During our analysis, we also randomly picked regions with the distribution of their sizes matched to that of REST peaks as controls.

### Identification of alignable peaks and conserved peaks

**Figure 1.**
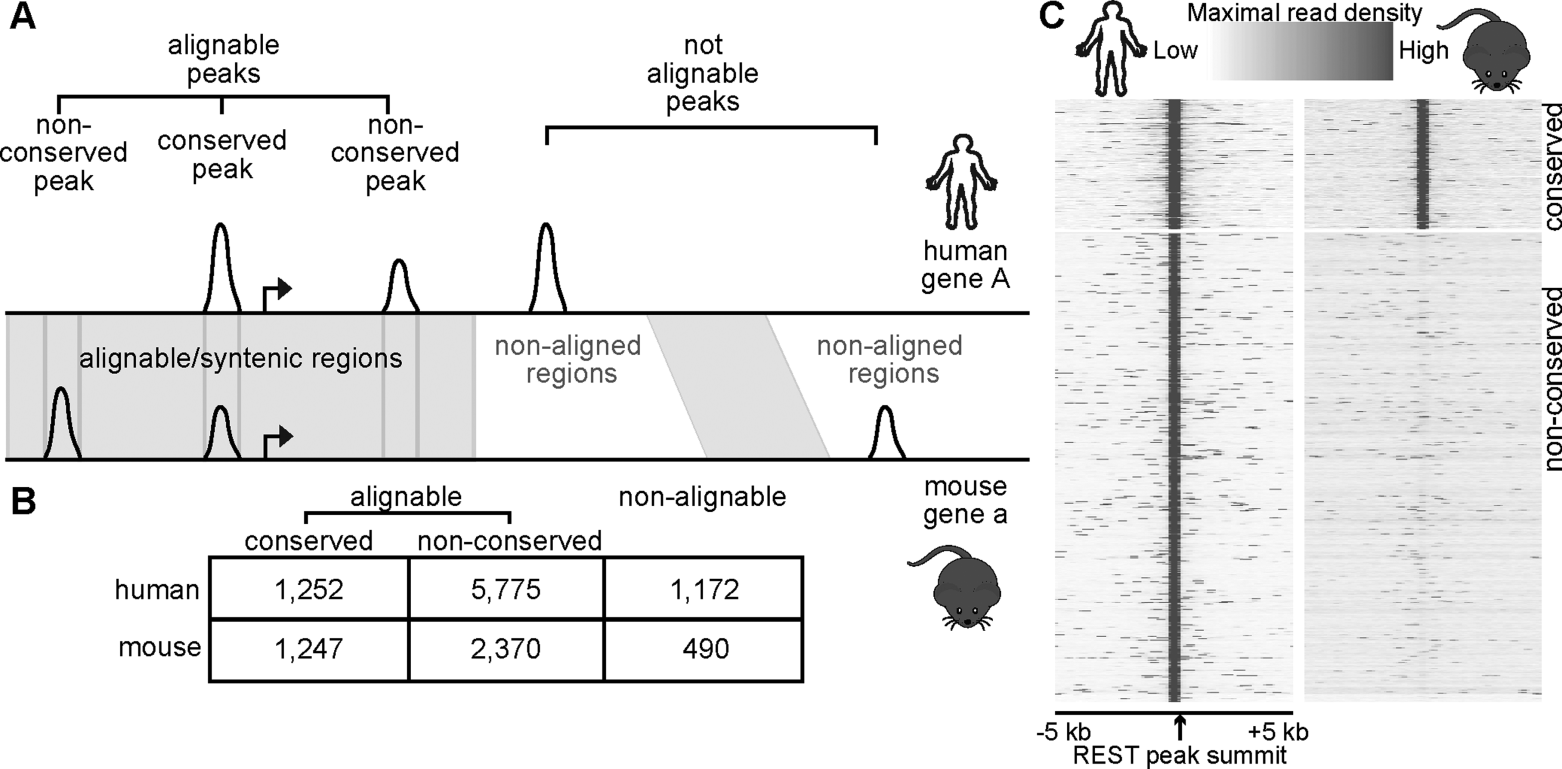
Human and mouse REST peaks and their synteny. (**A**) A cartoon illustrating the definition of alignable and conserved peaks. (**B**) Total numbers of REST peaks in individual groups. (**C**) Heatmaps showing ChIP-seq read densities in 50 bp bins from −5 kb to 5 kb of the summits of all conserved (top) and non-conserved (bottom) hESC REST peaks. Mouse data were centered on the syntenic summits of hESC peaks.

To identify syntenic regions in the human and the mouse genomes, we retrieved the locations and sequences of the Ensembl EPO multispecies genomic alignments (release 73) for 13 eutherian mammals (human- GRCh37, gorilla-gorGor3.1, chimpanzee-CHIMP2.1.4, orangutan-PPYG2, macaque- MMUL_1, marmoset-C_jacchus3.2.1, mouse-GRCm38, rat-Rnor_5.0, rabbit-oryCun2, horse-EquCab2, dog-CanFam3.1, pig-Sscrofa10.2, cow-UMD3.1) (38–40). We also used the UCSC's liftOver tool (41) to convert genome coordinates between assemblies, with the minMatch parameter set to 0.1, when converting peaks between humans and mice. A human ChIP-seq peak was considered alignable if ≥ 1 bp within it could be aligned to the mouse genome and *vice versa*. An alignable peak was marked as conserved if a peak overlapped with ≥ 1 bp of its syntenic region was also called in the other species (Figure 1). Note that we changed this ≥ 1 bp criterion to either more stringent or more tolerant and did not observe much difference in our results.

### RE1 motif analysis within REST peaks and assessment of RE1 motif conservation

We used MEME 4.6.1 (42) to find enriched motifs within the REST peaks, using 200 bp sequences centered on the peak summits. The top motif matched to known RE1 motifs, essentially identical to those previously identified (14,24,43), and the resultant position specific scoring matrixes (PSSMs) were used by the program MAST in the MEME suite to re-scan the 200 bp sequences for motif occurrence with default parameters. Almost all identifiable RE1 motifs lie within this region; very few RE1s were found in the next 200 bp. To assess conservation of RE1 motifs, we used MAST and the RE1 PSSMs to scan the entire peak for RE1 motifs and record the motif locations and scores and keep the one with the best score (*S*_human_). Then, we scanned the syntenic region of the selected RE1 site, with the scanning region restricted to no more than ±100 bp of the expected RE1 site (based on the motif distributions above and the fact that <3% of RE1 motifs moved >90 bp away from their syntenic positions), for short sequences matching to PSSMs. If the returned RE1 site with the highest PSSM score (*S*_other_) was similar enough to the human motif score (*S*_other_ >*S*_human_ × 0.8), we called these RE1s shared between humans and that species. If the RE1 was high scoring in any of the primate species (gorilla, chimpanzee, orangutan, macaque or marmoset) but not in any of the mammalian species (horse, dog, pig, mouse, rabbit, rat, cow) we called it primate-specific.

### Identification of genes with REST binding

We used the RefSeq (44) annotation from the UCSC genome browser (45) to define genes with REST peaks. An in-house python script assigned peaks to genes in this sequential order: to promoter regions (−5 kb to +1 kb of transcription start sites (TSSs)), to exons, to introns, to distal regulatory regions (−50 kb of transcription starts to +50 kb of transcription ends). When mapping peaks to either promoters or distal regions, only one gene with the closest TSS was selected. A single base overlap was used for these assignments. A peak can be mapped to multiple genes if it is equidistant from the TSSs of these genes or if it is located to exons or introns shared by these genes.

### Identification of orthologous genes

We used the Mouse Genome Informatics Mouse/Human Orthology dataset (ftp://ftp.informatics.jax.org/pub/reports/HMD_HumanPhenotype.rpt) to establish human-mouse orthologous relationship. To be more inclusive, we also considered genes with the same names in the Refseq annotations of the two genomes as orthologs.

### Base-level sequence conservation analysis

GERP scores were calculated at each base within ± 500 bp of peak summits (or the center of the peak if no summit was called, or the RE1 motif locations where specified) from the hg19 and mm9 GERP++ tracks data for base-wise scores, downloaded from http://mendel.stanford.edu/SidowLab/downloads/gerp/. To utilize the mouse data, mouse peaks were lifted back to mm9 using liftOver.

### Plotting GERP scores at REST occupied cRE1s

The GERP (46) scores for each peak were represented by a row in the matrix, with conserved peaks at the top, non-conserved in the middle and non-alignable peaks at the bottom. The resultant matrix was imported into Java TreeView (47) for coloring and visualization.

### Identifying clusters of co-factor/HM binding

The ChIP-seq read densities were calculated using the program seqMiner (48), which yielded for each peak an array of the maximal number of overlapping ChIP-seq reads (extended to 200 bp) in 50 bp bins from −5 kb to +5 kb of the peak summit. Within seqMiner the resultant density matrix for each group of peaks was clustered by k-means algorithm and then heatmaps were generated.

### DNA methylation analysis

Base-level DNA methylation data were downloaded from the GEO accessions/websites listed in Supplementary Table S1. Since the data were for hg18 and mm9, we used liftOver to lift ChIP-seq peaks to these two genome assemblies. Afterward, at each informative CpG within ± 5000 bp of REST peak summits, the number of all CpGs and the number of methylated CpGs were extracted from the data. To generate a smooth profile plot, we computed the average of the percent methylation across informative bases by sliding 10 bp windows. To find the significance of levels of methylation distal to and at the REST peak summit, we compared the methylation levels of all informative CpGs distal to the binding site with those at the binding site.

### DNA accession

All data are publicly available and they can be accessed in the Gene Expression Omnibus (see Supplementary Table S1 for accession information).

## RESULTS

### A core group of REST binding sites is conserved from humans to mice

We set out to explore the similarity and divergence of REST occupancy across species for better understanding REST functions. By reanalyzing previous published ChIP-seq data in embryonic stem cells (18,19,49) (Supplementary Table S1), we identified and characterized genome-wide REST binding sites (i.e. REST cistrome) in humans and mice. In order to obtain high-quality and reproducible REST binding information, we used the spp pipeline (35) to call peaks and the IDR method (36) to infer optimal thresholds from replicates. In the end, we identified about twice as many peaks (i.e. REST sites) in human ESCs (hESCs) as in mouse ESCs (mESCs) (*n* = 8199 versus 4107) (Supplementary Table S2). We believe that this expanded human REST binding reflects a true biological difference as opposed to experimental variables because the peak call results are robustly reproduced in replicates (Supplementary Figure S1A/B). Nevertheless, there are genes with strong REST peaks in mESCs only, for example, both *Lrrc61* and *Rabep2* promoters exhibited strong REST binding only in mESCs. REST can bind DNA sequences with a canonical 21 bp binding motif (referred to as the cRE1), non-canonical motif (ncRE1), half of the RE1 motif or no motif (Supplementary Figure S2) (14). Our motif analysis showed that similar percentages of human and mouse REST peaks were enriched with RE1 motifs, with 95.3% of hESC and 99.1% of mESC REST peaks containing either a cRE1, ncRE1 or a half RE1, respectively. This finding further supports the idea that more REST peaks in hESCs likely represent genuine expanded REST occupancy in humans (Supplementary Table S3). To investigate if any non-RE1-related motifs were enriched at the human-specific REST peaks, we use MEME-ChIP (50) to identify motifs. Interestingly, the top non-RE1 motif in human-specific peaks was for ASCL2 (Centrimo E-value < 1.4e-84), a basic helix-loop-helix TF involved in CNS development, while the top non-RE1 motif in conserved peaks belonged to E2F2, associated with cell cycle (Centrimo E-values < 1.0e-24).

Next, we utilized comparative genomic data to locate REST peaks that are present in syntenic (i.e. orthologous) genomic regions (Figure 1A). We considered data from two different sources for defining synteny between the human and the mouse genomes. First, we used the EPO multispecies alignment (MSA) (39,40) of 13 eutherian mammals from the Ensembl (38). Second, we employed human-mouse best aligned/longest syntenic regions file with liftOver (41), obtained from the UCSC genome browser (45). By individual methods, ∼70% of the REST peaks were located within the syntenically alignable genomic regions, and referred to as ‘alignable peaks’ (Supplementary Table S4). When combined, ∼85% of the REST peaks were in alignable regions (Figure 1A/B; Supplementary Table S4). Unless noted otherwise, all analyses described below were based on the genome alignment information merged from the EPO and liftOver data, resulting in ∼15% of ‘non-alignable’ REST peaks (Figure 1B).

Alignable REST peaks were further segregated into ‘conserved peaks,’ if peaks were called from ESCs of both species at the alignable/syntenic positions, or ‘non-conserved peaks’ (Figure 1; Supplementary Table S2). In the end, we found that 15.3% of human REST peaks were conserved in mESCs, whereas 30.4% of mESCs peaks were conserved in hESCs. The 15–30% conservation is consistent with a previous but limited study (30), which found that ∼25% of mESC REST peaks had conserved binding in human Jurkat T-cells, a very different cell type. Analysis of ChIP-seq read densities at mouse syntenic positions of human REST peaks confirmed that there was no enrichment of ChIP-seq signals in the mESCs (Figure 1C). Since there is no cross-species information to analyze for the non-alignable peaks, we decided to focus our comparative analysis on the alignable conserved peaks and the aligned non-conserved peaks. Regardless, we did not observe a significant difference in our findings if we expanded our definition of non-conserved peaks to include non-alignable peaks (data not shown).

### REST shows more conserved binding than ESC specific TFs

**Figure 2.**
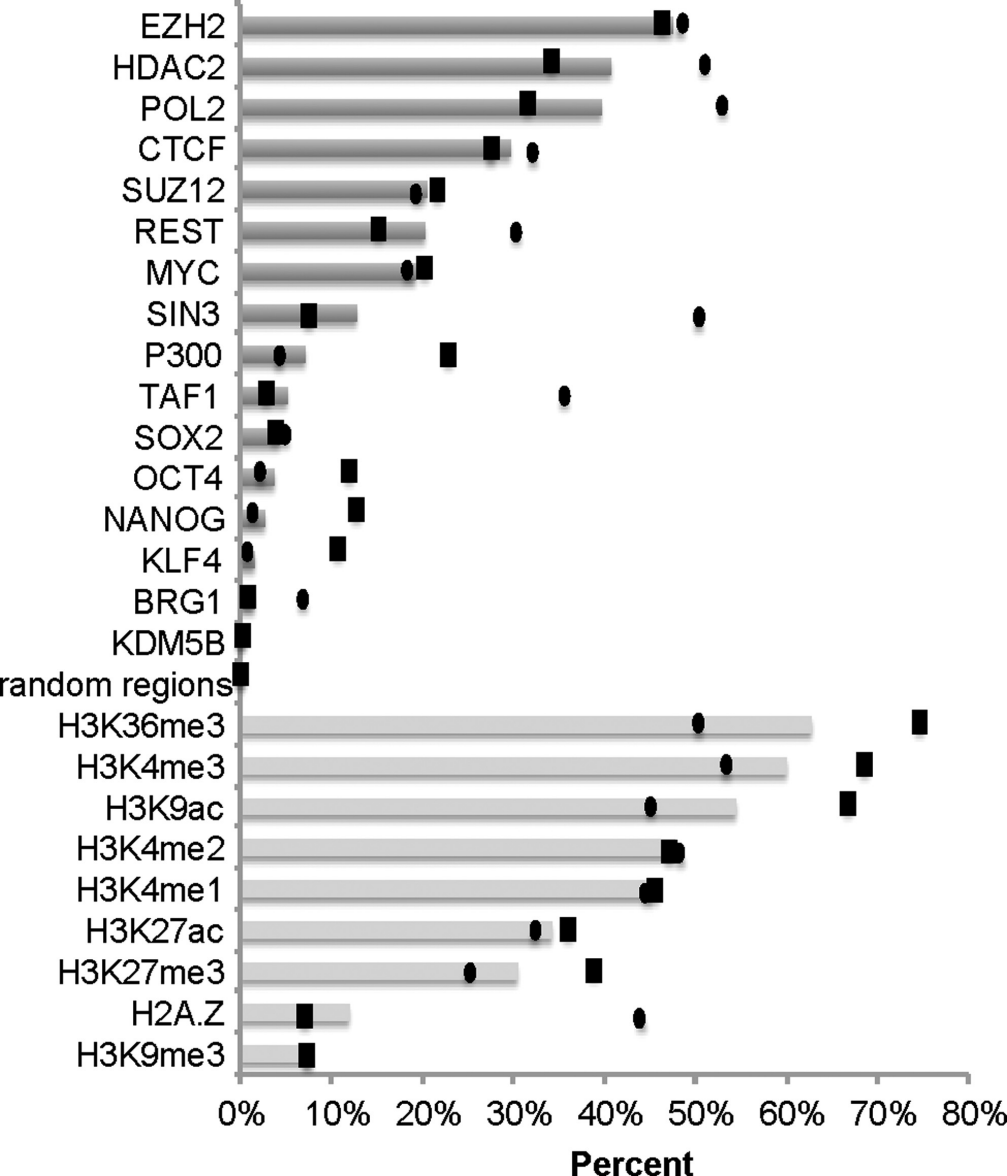
Percentages of conserved ChIP-seq peaks for TFs, co-regulators, chromatin modifiers and HMs. Bar plots show the ‘average’, defined as (*N*_human_conserved_ + *N*_mouse_conserved_)/(*N*_human_peaks_ + *N*_mouse_peaks_), a Jaccard similarity coefficient-like metric, in order to account for different numbers of peaks called for the two ESCs. Black boxes and ovals mark the actual conserved percentages observed for the hESCs and mESCs, respectively.

The low degree of conserved REST cistromes led us to wonder how REST compared to other TFs (including sequence-specific TFs, components of transcriptional machinery, e.g. TAF1 and PolII, chromatin modifiers, e.g. BRG1 and HDACs and co-regulators, e.g. SIN3). It has been suggested previously that TFs expressed in restrictive tissues (e.g. CEBPA and HNF4α) exhibited less conserved binding than more broadly expressed TFs such as CTCF (4,5). Our result from the analysis of 15 TFs and chromatin modifiers (Supplementary Tables S1 and S4) indicated that REST binding was strongly conserved, nearly as much as CTCF binding (27.5% of hESC CTCF peaks had conserved mESC peaks) and more than the bindings of other ESC TFs that are functionally restricted to ESCs, e.g. NANOG and OCT4 (Figure 2; Supplementary Table S4), co-regulators and chromatin modifiers. Relative to TFs, HMs displayed greater conservation between hESCs and mESCs, especially for promoter-associated marks (H3K4me3 – 68.6% and H3K9ac – 66.8%; Supplementary Table S4). These observations are similar to what have been reported previously (2,4–6,12,51). Note that the greater conservation of regions enriched with HMs could be partially explained by their broadness and gene content.

To better appreciate the cross-species binding conservation, we also asked how it compared to the shared REST occupancy across different cell types of the same species. A comparison of the REST peaks in hESCs to other human cell types (24) showed that 24.4–55.3% of hESC REST peaks were present in other cell types, suggesting that cross-species variation in REST binding is higher than the cell type binding diversity in the same species. Nevertheless, conserved REST peaks were more likely to be REST occupied across human cell types.

### Conserved REST binding occurs at genomic sites with strong sequence conservation

For the relationship of RE1 motif occurrence and REST peak conservation, we found that conserved REST peaks were more significantly enriched in cRE1 motifs (81.4%) than non-conserved peaks (53.4%), suggesting sequence conservation underlies highly conserved REST binding (Supplementary Table S3). As expected, conserved REST peaks tended to be at promoter regions (28.7% versus 13.3% of non-conserved peaks).

**Figure 3.**
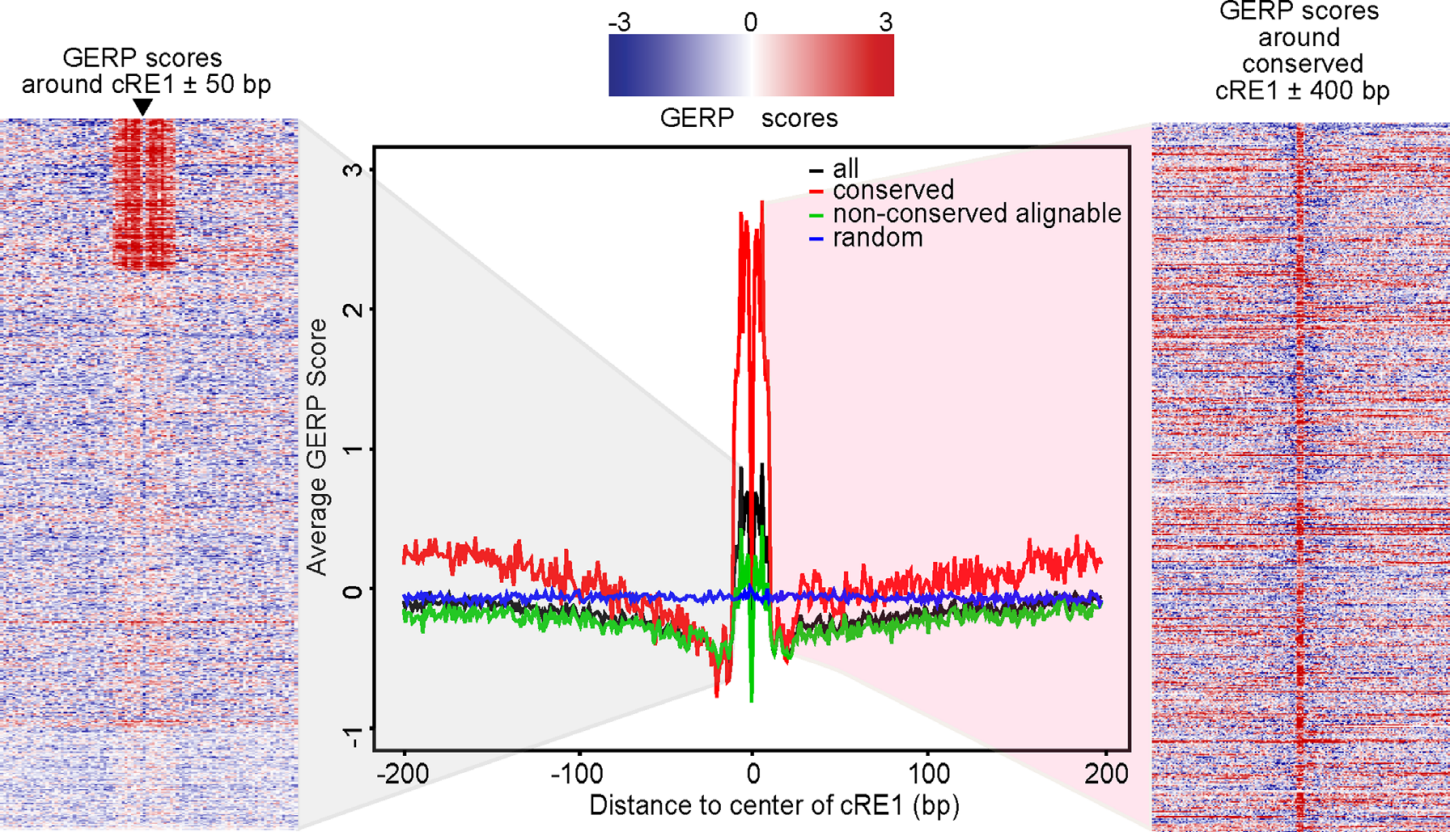
GERP scores at REST peaks. (Center) Profiles of GERP scores at hESC REST peaks. Y-axis shows the averaged GERP scores from GERP++ base-wise scores for all, conserved or non-conserved REST peaks, with random genomic regions as control. (Left) Heatmap of base-level GERP scores from −50 bp to 50 bp of the cRE1 in the cRE1 containing hESC REST peaks. Arrow marks the RE1 motif central bases with lower GERP scores. (Right) Heatmap of GERP scores from −400 bp to 400 bp of the cRE1 in the conserved hESC REST peaks.

To gain a better understanding of the sequence conservation at conserved REST peaks, we characterized base-level conservation using GERP scores (46). GERP identifies conserved base by quantifying the deviation of its observed changes across species from a neutral substitution rate, with high GERP scores indicating strong purifying selection. Since the RE1 motifs were enriched at the centers of REST peaks (data not shown), we decided to focus on sequence conservation within ±200 bp of the cRE1s in cRE1-containing REST peaks. As expected, we observed that conserved REST peaks had the highest sequence conservation, as supported by high GERP scores (Figure 3; center). Furthermore, the maximal GERP scores within the 21 bp of the cRE1s in conserved peaks were about 12× higher than the GERP scores of the flanking ± 50 bp sequences (Figure 3). This argues that the RE1 motifs at those REST binding regions are the most functionally important bases under strong evolutionary constraints.

### Non-conserved human REST peaks frequently contain primate-specific RE1s

While the conservation analysis underscored the importance of conserved RE1 motifs in determining human-mouse conserved REST binding, we also wanted to assess the evolutionary history of human-specific REST binding sites. We thus analyzed the evolutionary histories of RE1 motifs within the non-conserved human REST peaks. Interrogating the EPO alignment to compare putative RE1s among 13 eutherian mammalian genomes, we found that RE1 sequences in about half (50.5%, *n* = 1218) of the non-conserved and cRE1-containing human REST peaks (*n* = 2413) did not have a matching cRE1 motif in the mouse genome. Moreover, 27.9% (340/1,218) of those cRE1 containing non-conserved peaks had primate or human-specific RE1 motifs, indicating REST binds to many evolutionarily young RE1 motifs in hESCs, similar to previous report (30). Extending this analysis to non-conserved peaks containing more degenerate RE1s (ncRE1s and half RE1 motifs) (*n* = 1873), we found that 22.2% (*n* = 415) of the peaks had no identifiable motif in the mouse genome, 41.0% of which (*n* = 170) bound to a primate-specific motif. These results indicate that new RE1s explain a large portion of REST binding site expansion in the hominid lineage.

There have been reports that TEs rewire TF regulatory networks (3,5) and specifically that LINE2 (long interspersed nuclear element type 2) retrotransposition has been important for the birth of human-specific RE1s (29). We overlapped RepeatMasker's annotation of repetitive elements (http://www.repeatmasker.org) with hESC REST peaks. While a quarter (25.4%) of the peaks overlapped with the RepeatMasker-identified repetitive elements, most of the overlap was located at TEs (80.7%; *n* = 1680). Interestingly non-conserved peaks were more likely than conserved peaks to be enriched with TEs including long terminal repeats, LINEs, short interspersed nuclear elements (SINEs) and DNA transposons (1.3–3.0× more; binomial *P*-values < 4.4e-11), suggesting that each of these elements might have contributed to the primate specific RE1 expansion.

### Conserved REST binding is highly co-localized with selected transcription factors and HMs

As REST cooperates with several co-factors to determine local chromatin contents and confer gene regulation (26,27,52–62), we next decided to study the co-localization of REST and several TFs (*via* peak overlapping). Some of these proteins have been reported to interact with REST (HDAC1/2, SIN3, COREST, LSD1 and BRG1), while others (NANOG, OCT4 and SOX2) have been shown to indirectly co-regulate REST targets (17). We found that REST was frequently (>10% of REST peaks) and significantly (>3× more than expected by chance) co-localized (peak overlapping ≥1 bp) with SIN3 and HDAC2 in both human and mouse ESCs, while mESC REST peaks additionally were co-localized with two other chromatin modifiers: HDAC1 and P300 (Supplementary Table S5). On the contrary, TFs not known to interact with REST (e.g. NANOG and SOX2) did not exhibit significant co-localization with REST. Therefore, our results suggest that REST co-operates with a selective set of co-factors to regulate gene expression in both humans and mice.

We also analyzed local chromatin modifications at REST peaks and their conservation between humans and mice. Previous studies found that repressive HMs, such as H3K27me3, are highly enriched at REST binding sites (18,63), especially at gene promoters with cRE1s. By overlapping REST peaks with genomic regions enriched with HMs (≥ 1 bp), we found that besides H3K27me3 there was frequent and significant co-occurrence of REST with H3K4me2/3 and H3K9ac (Supplementary Table S5).

We next selected highly overlapping co-factors and HMs to study how co-factors and HMs cooperated at REST sites. We performed clustering analysis of REST peaks from the co-factor and HM ChIP-seq signals and revealed three types of REST cistromes: type I – co-localized with HDAC and SIN3 only; type II – co-localized with HDAC, SIN3, H3K4me2/3 and H3K9ac; and type III – not highly co-localized with any co-factors or HMs examined. Overall, 41.2% of mESC REST peaks could be classified as type I or type II (a similar percentage of human peaks was classified as type I or II). These included REST peaks in 14 of 15 well-characterized REST targets (24) in hESCs and 12 of 15 in mESCs. The lack of a cluster with the repressive H3K27me3 mark only and the relative abundance of type II peaks were unexpected, but they underscore the large array of co-factors that can cooperate with REST for regulating genes. The relative lack of REST-H3K27me3 association is, however, consistent with a recent finding from McGann*et al*. (64). We have previously reported high expression of REST-SIN3 targeted genes in human lymphoblastoid cells (24), suggesting that the quantity of type II peaks might be recapitulated in other cell types.

**Figure 4.**
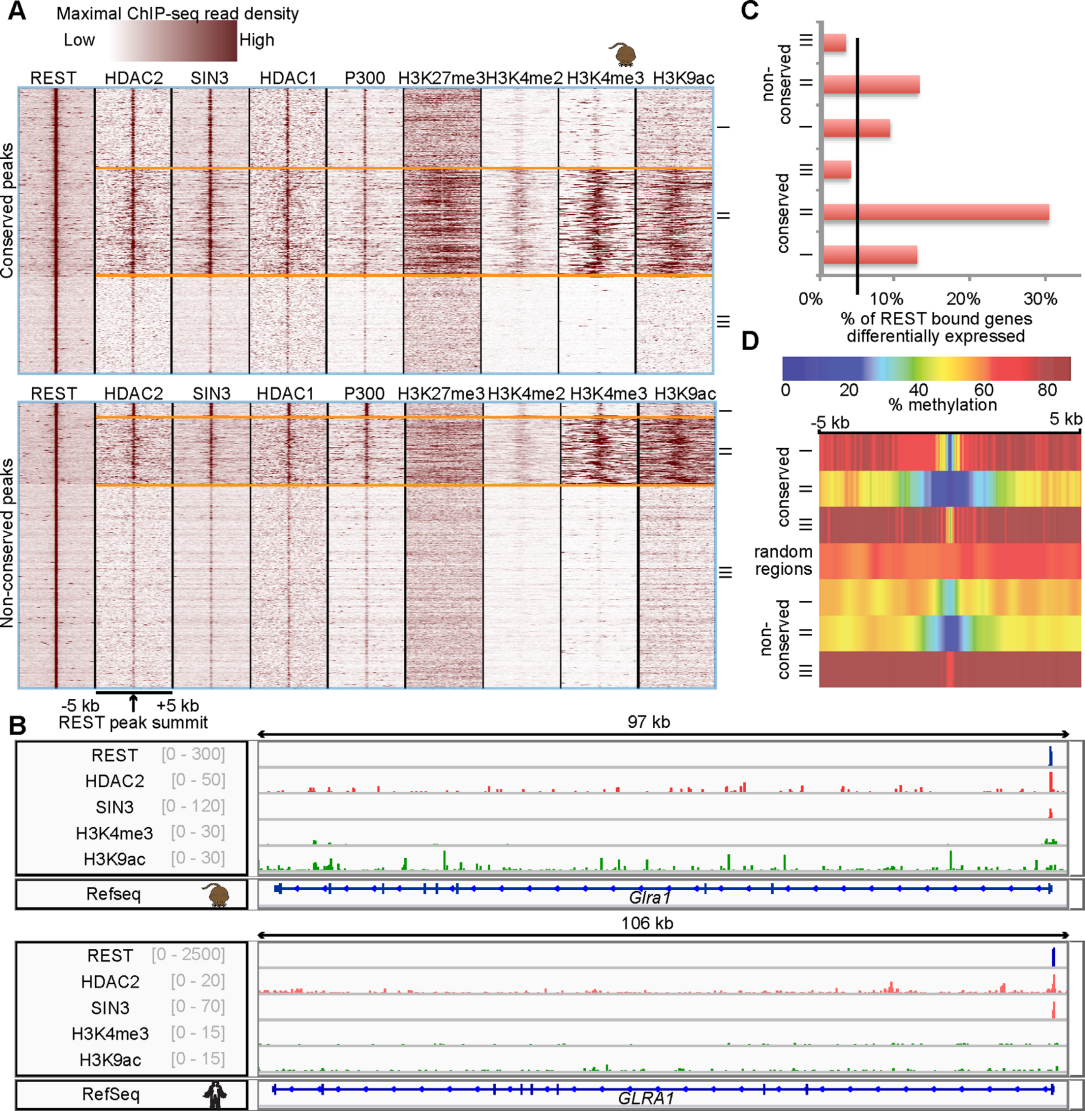
Clusters of REST peaks according to co-factors and HMs ChIP-seq data. (**A**) Heatmaps of maximal read coverages in 50 bp bins from -5 kb to 5kb of the mESC REST peak summits at conserved (top) and non-conserved (bottom) REST peaks. (**B**) Pileup of REST, co-factors and HM ChIP-seq reads at a type II REST peak in *GLRA1*, with preserved co-occurrence in both hESCs and mESCs, scale shown in gray. (**C**) Percentages of genes upregulated in mESCs upon REST KO (64). The black line marks% of upregulated genes at the genome-wide level. (**D**) Profiles of average CpG methylation percentage at REST peaks and flanking regions.

The co-localization of REST binding with HMs and co-factors was higher for conserved REST peaks than non-conserved peaks, as a greater fraction of conserved (Figure 4A, top) than non-conserved (Figure 4A, bottom) REST peaks were located in type I and II clusters (Supplementary Figure S3A; Supplementary Table S6). Indeed, many of the representative REST binding sites were co-localized with similar types of co-factors and HMs in both species (Figure 4B). Genome-wide analysis of co-factors and HMs also confirmed this (Table 1), with 51.4 and 83.3% of mouse type I and type II conserved REST peaks being classified as type I and II in hESCs, respectively. Type II peaks were preferentially localized to promoter regions (50% of human type II peaks in gene promoters), while type I and III peaks were preferentially localized to introns (53% of each). Surprisingly, motif analysis revealed that a greater percentage of type I peaks (75.1%, human) had cRE1 motifs than type II (47.2%) or type III (59.3%) peaks.

**Table 1.** Numbers and percentages of conserved REST peaks remaining the same type or changing between hESCs and mESCs

Type	hESC REST Peaks			mESC REST Peaks		
	Remain the same type in mESCs (*N*)	Change type in mESCs (*N*)	Remain the same type in mESCs (%)	Remain the same type in hESCs (*N*)	Change type in hESCs (*N*)	Remain the same type in hESCs (%)
I	179	166	51.9%	179	169	51.4%
II	398	85	82.4%	399	80	83.3%
III	294	130	69.3%	291	129	69.3%

Investigating non-RE1-related motif enrichment at the three types of human REST peaks, we found that ASCL2 was the top non-RE1 motif in type III peaks (Centrimo E-value < 5.4e-85). The top motifs in type I and II peaks were E2F motifs, associated with cell cycle (Centrimo E-value < 1.1e-11). This result is not entirely surprising as type III peaks are enriched in non-conserved REST binding and type I and II peaks are more enriched in conserved REST binding.

Additionally, functional analysis showed that the top pathways enriched with genes bound by type I REST peaks in human were metal ion, cation, ion and calcium ion transport (*P*-value < 1.46e-5), while the top pathways for type II and III human targets were neuron development and cell projection organization (*P*-value < 2.69e-11).

To study the effect of the different combinations of REST co-factors on target gene expression, we reanalyzed published human REST knockdown (KD) microarrays and mouse REST knockout (KO) RNA-seq expression datasets (two in HEK293, one in MCF10a and one in T47D human cell lines (65,66); one in mouse ESCs (64)) (Supplementary Tables S1 and S7). REST bound to 39.9% of the differentially expressed genes in REST KO mESC (3.4-fold more than expected, *P*-value = 2.1E-84). These direct targets were enriched in type I and type II peaks (Figure 4C, Supplementary Figure S3B). They were also more highly targeted by conserved peaks. These results suggest that REST binding with multiple co-factors and HMs (including active histone marks) is more likely to be functionally affected by REST occupancy.

### REST binding sites are generally hypomethylated in ESCs

Another type of epigenetic modification, DNA methylation, may also interact with REST and affect its function. While REST binding has been shown to be independent of the methylation status of the underlying RE1 sequences (67), MeCP2 is a component of the CoREST complex (67,68) and DNMT1 was found in the RE1 regions of some neuronal genes (69). Moreover, the Schübeler group found that TFBSs had low methylation in mESCs (70,71) and methylation reduction depended on the presence of TFs, like REST. Using previously published bisulfite sequencing (Bis-seq) data in human and mouse ESCs (70–72) (Supplementary Table S1) to compare DNA methylation level at REST peaks and their flanking ± 5 kb regions, we observed that mESC and hESC REST peaks were hypomethylated (Figure 4D, Supplementary Figure S3C), consistent with previous findings (70). Although promoters were generally less methylated than gene bodies, we found that REST peaks in both locations were hypomethylated relative to their flanking regions. When co-factors and HMs were considered, we found that both type I and type II peaks were more hypomethylated relative to their flanking regions than type III peaks or randomly chosen regions. This hypomethylation was accentuated in both depth and breadth at conserved peaks. These results demonstrate that selected types of REST binding are more correlated with low methylation regions.

**Figure 5.**
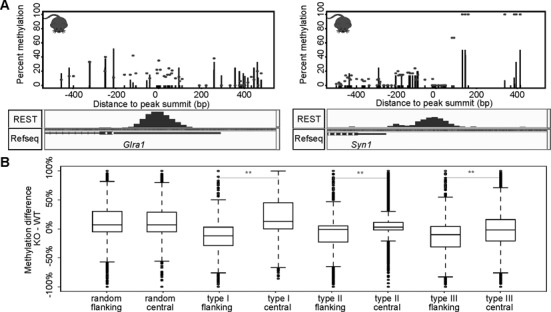
Change of DNA methylation at REST binding sites in REST KO mESCs. (**A**) Percentage of CpG methylation at informative CpGs in WT (black lines) and REST KO (black dots) in two genes: *Syn1* and *Glra1* with REST binding (bottom, ChIP-seq read density). (**B**) Boxplots of methylation difference between KO and WT experiments at all CpG sites within the REST peaks (central regions) and their flanking regions (a peak length ±5 kb from the REST peak summit). Asterisks mark significant differences in methylation change: *P*-value < 0.001.

When Bis-seq data in mESCs with REST KO (Supplementary Table S1) (71) were analyzed, we found that CpGs near several neuronal REST targets became hypermethylated upon REST KO (Figure 5A). It should be noted that there was a global reduction of DNA methylation upon REST KO, as reported by the original authors (71), although the mechanism is unclear. Nevertheless, in accordance with our above observations of hypomethylation at REST peaks, we found that CpGs within all three types of REST peaks showed significantly increased DNA methylation at the peak summit in the KO samples relative to the CpGs at regions adjacent to REST peaks (Figure 5B). This held in REST sites located to either promoter or gene bodies (data not shown). The difference was more prominent for type I peaks than for the other two types.

### There is expanded REST occupancy at many neuronal genes in humans

The above analyses demonstrated that in both humans and mice less than one-third of REST peaks in ESCs had conserved binding, but that REST conservation was highly associated with functional features: increased base-level conservation, presence of an RE1 motif and co-occurrence of co-factors, HMs and DNA methylation. At the gene level, we found that about a third (*n* = 1485) of human REST target genes were bound by a conserved REST peak or that their mouse homologs contained a REST peak. Pathway analysis of all REST-bound genes in hESCs or mESCs, conserved targets or genes with human-specific binding (Table 2; separated into genes with expanded binding in hESCs and genes with peaks only in hESCs) identified enrichment of pathways involved in neuronal functions. Overall, the enriched pathways were similar to what we previously reported for REST targets in 15 non-neuronal human cell types (24), suggesting that REST binding at many important neuronal genes is conserved across species and cell types.

**Table 2.** Enriched pathways in genes with REST-binding in humans and mice compared to genes with species-specific REST binding. Numbers refer to FDRs

GO: Biological Process	Human genes with peaks in both ESCs	Mouse genes with peaks in both ESCs	Genes with expanded binding in hESCs	Genes with expanded binding in mESCs	Genes with peaks only in hESCs	Genes with peaks only in mESCs
Neuron differentiation	1.23E-18	1.68E-14	2.23E-10	N.E.	N.E.	N.E.
Neuron development	6.70E-15	7.55E-10	4.62E-09	N.E.	N.E.	N.E.
Cell morphogenesis involved in neuron differentiation	4.00E-13	1.49E-10	1.57E-08	N.E.	N.E.	N.E.
Transmission of nerve impulse	2.00E-13	3.73E-12	1.45E-19	N.E.	N.E.	N.E.
Cell morphogenesis involved in differentiation	9.99E-13	2.46E-11	9.73E-08	N.E.	N.E.	N.E.
Cell-cell signaling	1.79E-12	2.55E-12	1.13E-18	N.E.	N.E.	N.E.
Synaptic transmission	2.39E-12	2.55E-12	3.94E-18	N.E.	N.E.	N.E.
Neuron projection development	8.56E-12	1.35E-09	1.02E-08	N.E.	N.E.	N.E.
Axonogenesis	7.96E-12	7.77E-09	6.18E-07	N.E.	N.E.	N.E.
Neuron projection morphogenesis	9.14E-11	2.02E-08	1.44E-07	N.E.	N.E.	N.E.
Cell projection organization	1.22E-09	2.13E-10	9.98E-09	N.E.	N.E.	N.E.
Metal ion transport	5.13E-10	7.46E-12	1.89E-13	N.E.	N.E.	N.E.
Ion transport	6.03E-08	6.70E-10	1.89E-13	N.E.	N.E.	N.E.
Learning or memory	4.46E-02	5.59E-02	2.98E-02	N.E.	5.64E-03	N.E.

N.E.: No reported enrichment.

Nevertheless, the large majority of REST binding is species specific. We decided to study the ‘human-specific’ aspects of REST occupancy. In total, REST peaks were proximal or distal to 4480 genes in hESCs, 1729 more than in mESCs. Most (*n* = 2995) of the hESC REST bound genes were not targeted by REST in mESCs. Products from some of these genes were predicted to locate to synapse (*n* = 64, *P* < 0.001) and axon (*n* = 31, *P* < 0.01). By Gene Ontology (GO) analysis (73), these human-specific REST bound genes were enriched in biological processes involved in learning or memory and positive regulation of transcription from the RNA polymerase II promoter (top terms – all with false discovery rate (FDR) < 0.05). KEGG pathway and ingenuity pathway analysis (IPA) of these genes also reported enrichments of neuronal pathways including axon guidance, GNRH signaling, CREB signaling in neurons and CRH signaling. Interestingly, CREB signaling has been implicated as acting in opposition to REST regulating several REST targets and REST itself (74). In addition, many genes (especially neuronal genes) also exhibited expanded human-specific REST binding.

Genes bound by REST in both species (*n* = 1341), were bound on average by two REST peaks in hESCs as opposed to one (1.4 on average) peak in mESCs. For example, *AUTS2* and *NRXN1–3* have 3–16 more peaks in hESCs than mESCs. Specific kinds of TEs were enriched in the expanded REST binding sites, relative to all REST peaks and randomly selected regions, including LINE2 and SINE/Alu elements (1.1× and 1.3× more, binomial *P* < 0.05), while SINE/MIR elements were relatively depleted (2.2× less, *P*-value = 5e-4). Genes with human-specific TE-associated REST peaks were also enriched in neural functions including transmission of nerve impulse, synaptic transmission and neuron projection development (*P* < 1e-3). Genes with REST binding only in mESCs (*n* = 1,333), however, were distinct and involved in signal transduction not specifically related to neural functions. This suggests that REST transcriptional networks may have been rewired extensively between humans and mice, leading to species-specific roles.

We were particularly intrigued by the presence of human-specific REST targets that are bound by REST not only in hESCs but also in all non-neuronal cell types for which we have ChIP-seq data. These genes (*n* = 201) are strong candidates for novel REST targets in humans or primates. Interestingly, they were enriched in mineralocorticoid biosynthesis and glucocorticoid biosynthesis (*P*-value < 2.2e-3) based on IPA. Some of these genes were neuronal and associated with CREB signaling in neurons, synaptic long-term potentiation (e.g. *GRIA4* and *CAMK4*), or learning or memory functions (e.g. *ADCY8* and *APP*). Note that about a third (*n* = 69) to a fourth (*n* = 49) of these genes were also bound by REST in SH-SY5Y cells (75) and neurons (24), suggesting that REST regulates this subset of genes in neurons and contributes to neuronal homeostasis and functions.

### Amyotrophic Lateral Sclerosis and oxidative stress genes are enriched in non-conserved human REST targets

**Figure 6.**
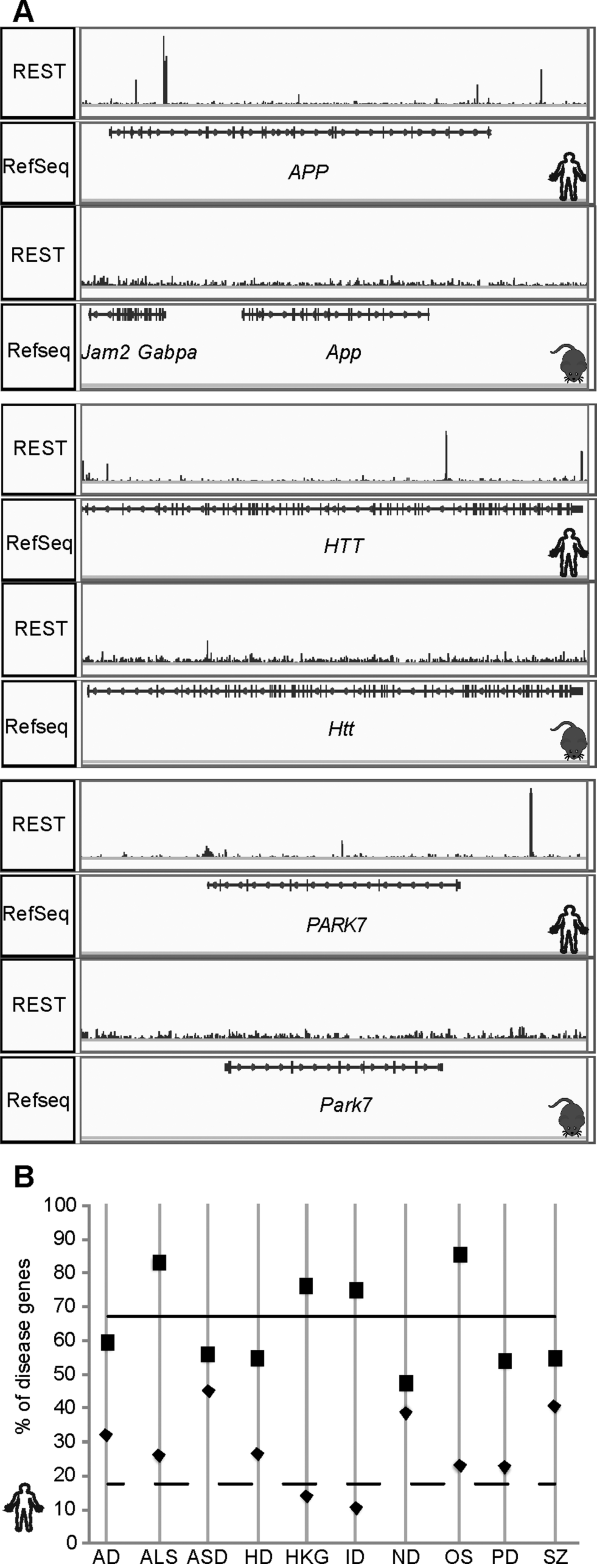
Selected disease-associated genes and their relationship to human-specific REST occupancy. (**A**) Pileup of REST ChIP-seq reads in hESCs and mESCs at three well-characterized disease causal genes: *APP/App, HTT/Htt, PARK7/Park7*. (**B**) Percentages of genes in disease-associated gene sets with any REST peaks (boxes, solid line) and percentages of REST-bound genes with peaks in hESCs only (diamonds, dashed line). The two lines represent genome-wide numbers.

The enrichment of genes for learning and memory functions among the human-specific REST-bound genes prompted us to study whether those genes with human-specific binding are related to brain disorders. This is especially interesting in light of a recent report that REST has a protective effect on Alzheimer's disease (AD) and mild cognitive impairment in aging brains (75), as well as several studies documenting that REST plays a role in neurodegenerative and neurodevelopmental diseases including Huntington's disease (HD), Down syndrome and schizophrenia (SZ) (76–82). First, we noticed that several well characterized causal genes for AD (*APP, PSEN2* and *SORL1*), HD (*HTT* and *SLC2A3*), and Parkinson's disease (PD) (*PARK2, PARK7*) were bound by REST in hESCs but not mESCs (Figure 6A). Moreover, *APP* was upregulated when REST was KD in human MCF10a cells, whereas its expression did not change in REST KO mESCs (Supplementary Table S7), suggesting potential functional implications for human REST binding to *APP*. Additionally, we identified 12 disease risk SNPs from the GWAS catalog (83) that overlapped with REST peaks. Almost all (*n* = 11) had non-conserved REST binding. Additionally, three were associated with neurological disorders or functions including SZ (rs4129585 at *TSNARE1*) (84), multiple sclerosis (rs12644284 at *TRIM2*) (85) and brain structure (rs12479254 at *BOK*) (86).

Therefore, we investigated whether genes implicated in brain disorders are more likely to be targeted by REST specifically in humans. From various databases, we obtained lists of genes associated with idiopathic forms of AD, HD, PD, SZ, autism spectrum disorders (ASD), Amyotrophic Lateral Sclerosis (ALS), intellectual disabilities (ID), neurodegenerative diseases (ND) and oxidative stress (OS), which has been linked with a variety of brain disorders (87–92), as well as housekeeping genes (HKG) as a control (93) (Supplementary Table S8). All of brain disorder gene sets except ID genes were enriched with REST binding relative to all human genes – probably due to the enrichment of neural genes in REST targets. Interestingly, only OS and ALS gene sets were enriched with both REST binding and human-specific REST binding (Figure 6B; Supplementary Table S9). None of the OS genes were in the set of ALS genes (Supplementary Figure S4). These results suggest that REST, by regulating newly emerged human-specific targets, may have novel, emerging and important roles in brain functions and disorders. Interestingly, REST's previously suggested role in OS response (75) may be human specific; as may be its potential role in ALS. For the six ALS and 13 OS genes with human-specific REST binding (Supplementary Table S9), we note that REST binding was also found in multiple human cell types (24). Additionally, REST peaks can be found at some of these genes in H1 ESC-derived neurons (24) and SH-SY5Y cells (75), with REST binding to *FUS* and *MAPK14* in both; to *DUSP4, DUSP6* and *MAPK10* only in neurons; and to *MAPKAPK2* only in SH-SY5Y cells. Moreover, examination of REST binding in two additional mouse cell lines (C2C12 mouse myoblast cells and neural progenitor cells), where ChIP-seq data were available, further confirmed the absence of REST binding in mice, with the exception of two conserved peaks in *Nefh* and one *Mapk10* peak that existed in mESCs but not within syntenic regions. Interestingly, four of the REST peaks proximal to ALS genes and two proximal to OS genes overlapped with TEs, further suggesting that TE-mediated expansion of RE1-like motifs may underlie human-specific REST binding and contribute to human-specific REST regulation. This is in line with recent reports that TE-mediated events may have critical roles in normal human brain functions and brain disorders (94,95).

## DISCUSSION

In this study, we have performed comparative analysis of REST binding in human and mouse ESCs and studied the potential biological implications of our findings. Overall, our results point to significant differences between the REST regulatory networks in humans and mice, underscoring the divergence of transcriptional networks between the species.

At the level of REST-chromatin interactions, we observed that most REST binding events are markedly different between hESCs and mESCs. However, at the gene and pathway level, we noted that both conserved and non-conserved hESC REST binding events targeted genes implicated in neural development and functions. Does this mean that identical (or similar) regulatory outcomes can be achieved from one TF as long as some key genes (not necessarily the same set of genes) in a functional pathway are targeted? Or does it suggest that core conserved REST targets play determinative roles while non-conserved ones have subsidiary roles in the dynamics and robustness of REST regulatory networks? The enrichment of conserved REST binding sites among differentially expressed genes upon REST disruption seems to suggest the latter. However, future experiments specifically designed to address these important issues will be required. On the other hand, numerous studies have documented the difficulties in using model organisms such as rodents to model human brain development and NDs (80,96–99). Perhaps the differential wiring of transcriptional regulatory networks, including the one mediated by REST, could be an important factor. In the case of Huntington's disease, it will be interesting to study whether the human-specific REST binding site in *HTT* contributes to any functional difference between the human *HTT* and the mouse *Htt* genes. In light of human-specific REST binding to several OS genes and the role of OS in neurodegenerative disorders (100), it is possible that REST has expanded roles in protecting aging human brains that do not exist in mouse brains. Alternatively, it is possible that additional levels of REST regulation are required to maintain the fidelity and ontogeny of human adult neurogenesis, as adult neurogenesis in the human brain is more dynamic and lasts longer than in the mouse brain (101). It is important to note that REST expression is highly correlated with cognitive preservation and longevity during aging (75), two processes that are very distinct between humans and mice.

There are several limitations in the current study. First, the analysis was carried out on data from ESCs only. While both hESCs and mESCs are at ground state of cell differentiation and development, they have some notable differences (102). It will be extremely informative to carry out our analysis utilizing differentiated cell types at various developmental stages to address whether REST regulatory networks become more or less similar between humans and mice. Additionally, determination of the number of loci occupied by a TF from ChIP-seq data is dependent on a number of factors including antibody quality, genome mappability, read depth and sequencing data quality, but we believe these factors have contributed little to our results. Finally, the 2× difference in the number of REST peaks between the two ESCs has made it difficult to compare statistics directly. Howeve,r we should emphasize that this difference did not have much effect on our conclusions, as 16.6% of hESCs would become conserved if ∼8000 REST peaks were called for mESCs, to match the peak numbers in hESCs.

In the analysis of sequence conservation, we identified a region of lower sequence conservation (i.e. conservation valley) proximal to the REST motif (Figure 3, Supplementary Figure S5). This valley of lower conservation encompassed ∼20 bp on either side of the motif. This phenomenon was reported previously from an analysis of REST binding sites (17) (see Figure 4E in (17)) and at Drosophila Twist TF binding sites (103) (see Figure 1A in (103)). We carried out similar analysis for the peaks of other TFs (CTCF, CEBPA, HNF4α) and HMs (H3K27ac, H3K27me3) and detected high-nucleotide conservation at peak summits for TFs but not HMs. However, the conservation valley was observed only for CTCF (Supplementary Figure S5). These observations suggest that the conservation valley might be limited to TFs such as REST and CTCF, both widely expressed with long DNA binding motifs. It will be interesting to study in the future if the valley is related to co-factor spacing.

Our study identified a group of REST binding sites (type II) that were co-occupied by known co-factors, including SIN3 and HDAC2, and active HMs. Our previous study has shown that many REST sites with SIN3 co-binding were localized to highly expressed genes (24). The fraction (24%) of type II REST peaks seems to be higher in ESCs than in more differentiated cell types, as 15 and 17% of REST peaks in GM12878 and Hep G2 (both human cell lines) are type II. This result may be related to more pervasive gene expression and greater chromatin plasticity of ESCs. Nevertheless, we should also mention that in cervical adenocarcinoma cells SIN3 was found at promoters marked with H3K4me3 (104), and in mouse neural stem cells H3K9ac or H3K4me3 were detected around the RE1 elements of 5/8 genes assessed (27). It is interesting that genes with type II REST peaks were more likely to be upregulated upon REST depletion. Nevertheless, it seems counterintuitive that active HMs were present in REST peaks, since REST principally represses gene transcription (105). Future studies will be required to address the actual order of occurrence of HMs and REST occupancy, as well as how HMs change at REST-bound regions when REST expression is reduced. It is possible that H3K4 methylation and H3K9 acetylation provide open chromatin regions for REST binding, as for HSF (106). Likewise, more studies will be needed to understand if hypomethylation at REST sites are from exclusion of DNA methyltransferases, active recruitment of demethylases by REST or other mechanisms.

## SUPPLEMENTARY DATA

Supplementary Data are available at NAR Online.

SUPPLEMENTARY DATA
