# Supplementary material for: Significant expansion of the REST/NRSF cistrome in human versus mouse embryonic stem cells: potential implications for neural development
Source: Nucleic Acids Res. 2015 May 18;43(12):5730–43. doi: 10.1093/nar/gkv514 (PMC4499139; doi:10.1093/nar/gkv514)
Supplement: SUPPLEMENTARY DATA [file supp_43_12_5730__index.html]

Significant expansion of the REST/NRSF cistrome in human versus mouse embryonic stem cells: potential implications for neural development — Significant expansion of the REST/NRSF cistrome in human versus mouse embryonic stem cells: potential implications for neural development — SUPPLEMENTARY DATA 

# Significant expansion of the REST/NRSF cistrome in human versus mouse embryonic stem cells: potential implications for neural development

## SUPPLEMENTARY DATA

- SUPPLEMENTARY DATA
- SUPPLEMENTARY DATA
