# Supplementary material for: Significant expansion of the REST/NRSF cistrome in human versus mouse embryonic stem cells: potential implications for neural development
Source: Nucleic Acids Res. 2015 May 18;43(12):5730–43. doi: 10.1093/nar/gkv514 (PMC4499139; doi:10.1093/nar/gkv514)
Supplement: SUPPLEMENTARY DATA [file supp_gkv514_nar-00580-h-2015-File008.pdf]

Significant Expansion of the REST/NRSF Cistrome in Human versus Mouse Embryonic Stem Cells: Potential Implications for Neural Development

Shira Rockowitz [1], Deyou Zheng [1,2,3,*]

1. *Department of Genetics, Albert Einstein College of Medicine, Bronx, NY 10461, U.S.A.*
2. *Department of Neurology, Albert Einstein College of Medicine, Bronx, NY 10461, U.S.A.*
3. *Department of Neuroscience, Albert Einstein College of Medicine, Bronx, NY 10461, U.S.A.*

*To whom correspondence should be addressed.

Tel: +1 718 839 7215

Fax: +1 718 430 8785

Email: deyou.zheng@einstein.yu.edu

SUPPLEMENTARY DATA

Supplemental Figures

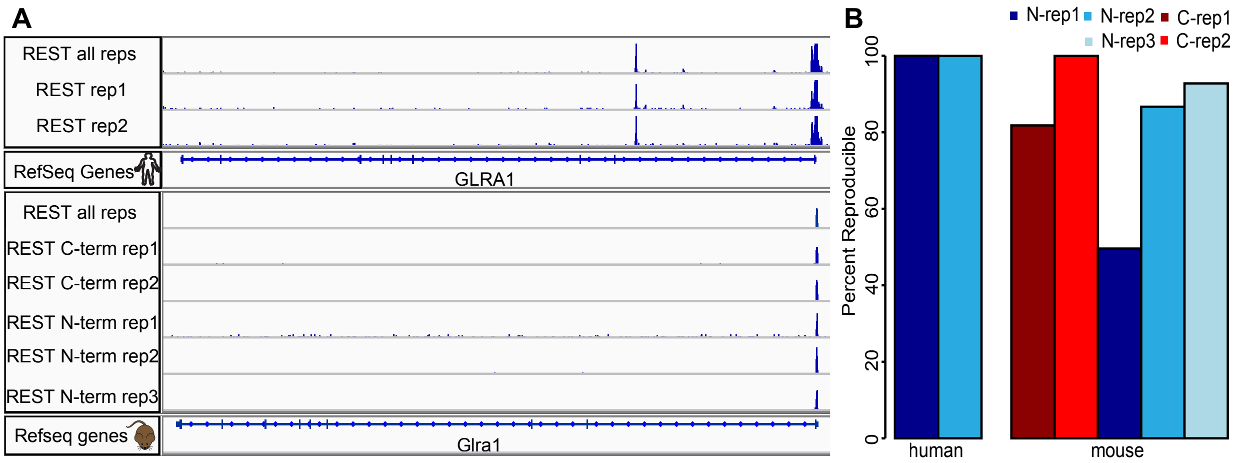

Figure S1. Reproducibility of REST binding across replicates. (A) Pileup of REST ChIP-seq reads across replicate REST ChIP-seq samples in hESCs and mESCs at *GLRA1/Glra1*. The top track is from pooled data and the bottoms are individual experiments. (B) Percentage of final peaks that was also called in each of the ChIP-seq replicate samples. “N” and “C” replicates (reps) indicate data from antibodies recognizing N- or C-terminal domain of REST protein, respectively.

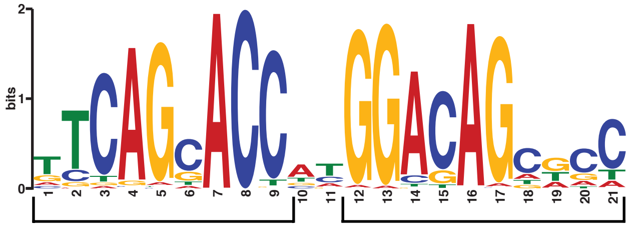

Figure S2. RE1 motif identified in all hESC ChIP-seq peaks. Lines demarcate left and right half sites of the motif.

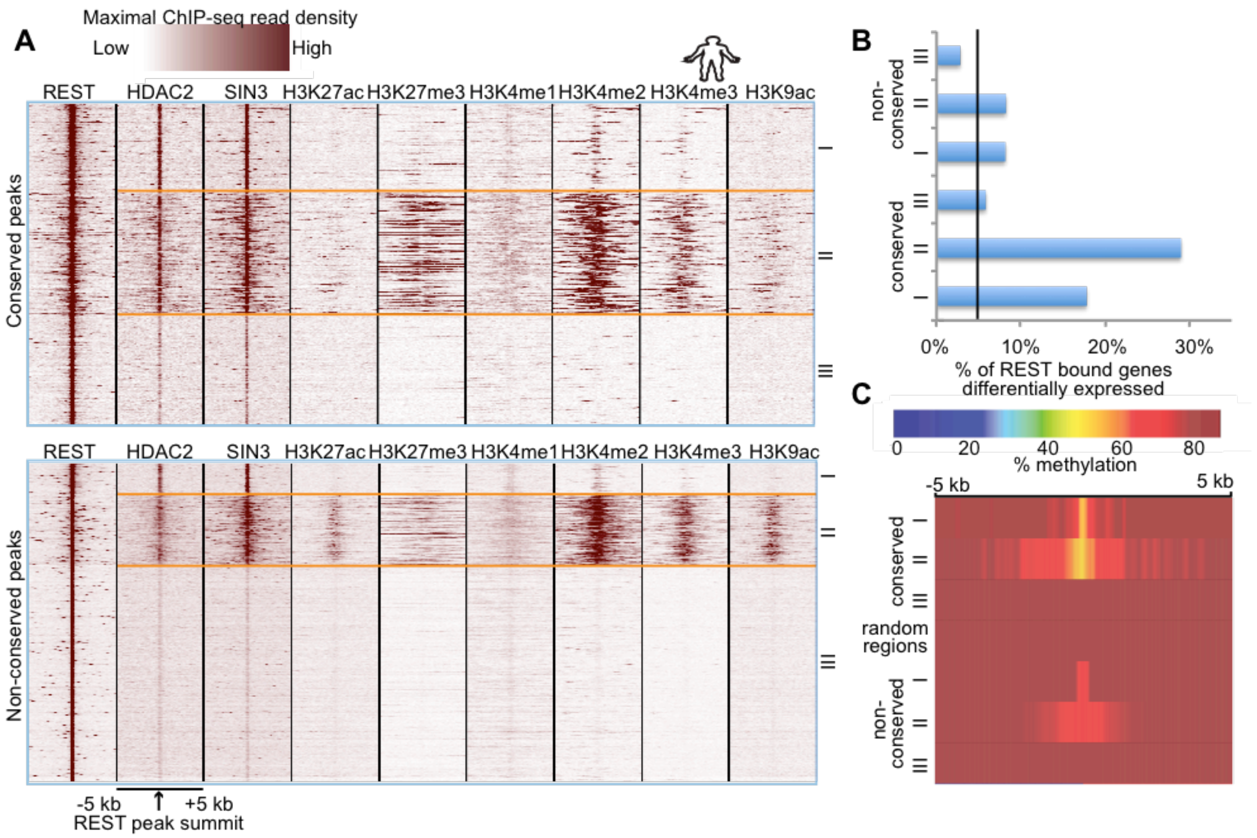

Figure S3. Human REST cistrome association with DNA hypomethylation and differential gene expression. (A) Heatmaps of maximal read coverages in 50 bp bins from -5 kb to 5kb of the hESC REST peak summits at conserved (top) and non-conserved (bottom) REST peaks. (B) Percentages of genes up-regulated in hESCs upon REST KD. The differential expression for human genes was complied from REST KD in non-ESCs as no REST KD/KO expression data were available from hESCs for the current analysis. For human data we compiled a list of upregulated genes from four REST KD experiments: two HEK293 (80, 81), an MCF10a (81) and an T47D (81) experiment. See Table S7 for a complete list of upregulated genes across all human and mouse REST KD/KO experiments. The black line marks % of up-regulated genes at the genome-wide level. (C) Profiles of CpG methylation percentage at REST peaks and flanking regions.

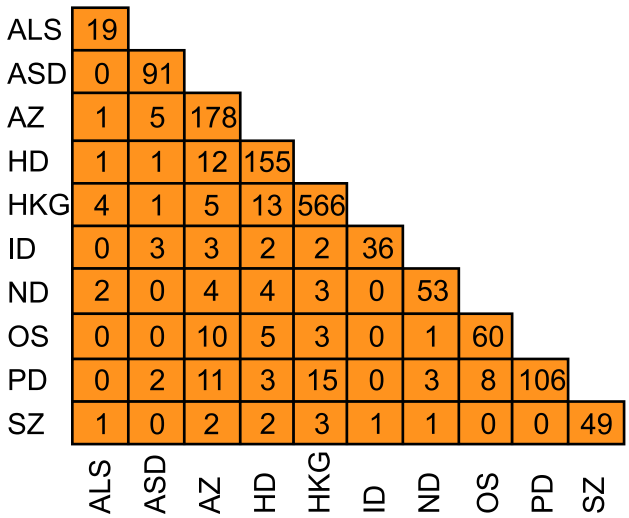

Figure S4. Overlap of gene sets used for disease-associated analysis.

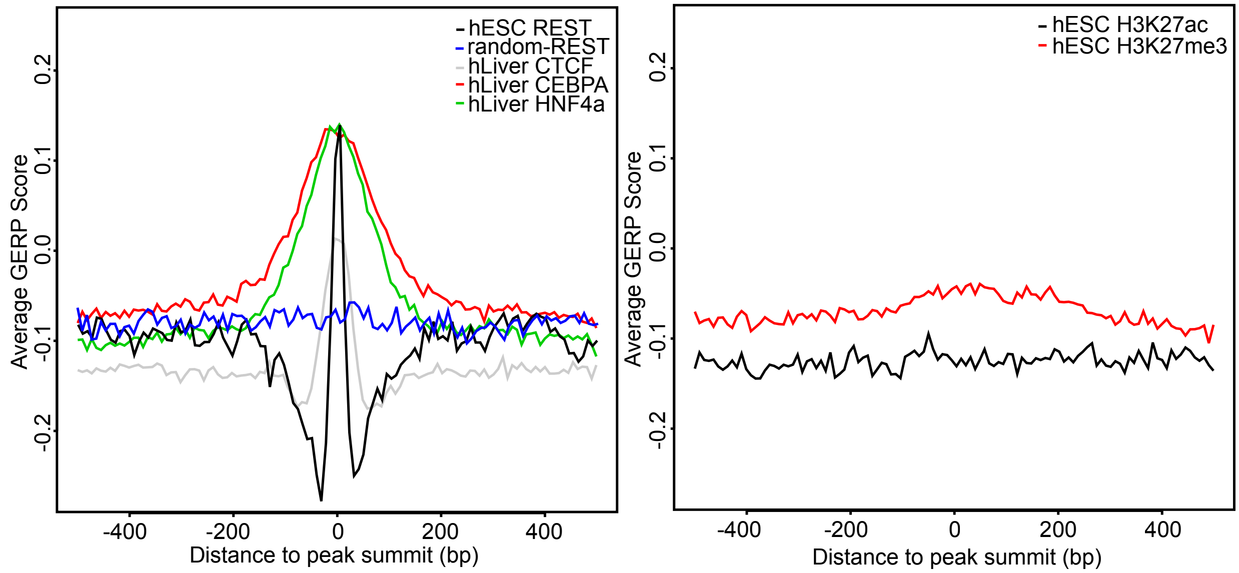

Figure S5. GERP scores at regions bound by selected TFs and or enriched with selected HMs. Y-axis shows the averaged GERP score from GERP++ base-wise scores (46) averaged over all peaks, from -500 bp to 500 bp of the peak summits. The HNF4α, CEBPA, and CTCF peaks were called from human liver ChIP-seq data, while the histone modifications were from hESCs.

Supplemental Tables

Table S1. **List of accession numbers and total reads of the ChIP-seq and expression datasets.**

See separate Excel file.

Table S2. List of all REST peaks identified in hESCs and mESCs, their relationship to genes, and their conservation. See separate Excel file.

Table S3. % of human and mouse, conserved and non-conserved REST peaks with RE1 motifs.

	human all	human conserved	human non-conserved	mouse all	mouse conserved	mouse non-conserved
**cRE1**	58.4%	81.4%	53.4%	66.6%	80.9%	61.2%
**ncRE1**	3.4%	5.4%	2.9%	4.0%	6.3%	2.9%
**half site**	33.6%	12.9%	37.8%	28.4%	12.7%	34.7%
**noRE1**	4.7%	0.3%	5.9%	0.9%	0.1%	1.3%
**promoter**	15.6%	28.7%	13.3%	18.4%	30.0%	13.3%
**exon**	5.9%	9.0%	5.4%	7.4%	7.5%	7.4%
**intron**	37.1%	32.4%	37.9%	35.0%	31.3%	36.6%
**distal**	20.6%	16.9%	21.3%	23.0%	17.3%	25.5%
**intergenic**	20.7%	13.1%	22.1%	16.2%	14.0%	17.2%

Table S4. Numbers and percentages of alignable and conserved peaks by different methods.

See separate Excel file.

Table S5. Percentages and Enrichments REST peaks colocalized with TFs and HMs.

	Percent of REST peaks with TF/HM, human	Fold Enrichment over random regions,	Percent of REST peaks with TF/HM, mouse	Fold Enrichment over random regions,

		human		mouse
	Transcription Factors			
**COREST**	N/A	N/A	3.4%	69.00
**LSD1**	N/A	N/A	4.7%	32.50
**KDM5B**	0.1%	0.00	0.7%	14.50
**SUZ12**	0.3%	3.14	0.9%	8.75
**HDAC6**	6.8%	3.91	N/A	N/A
**EZH2**	0.6%	4.18	0.6%	12.00
**SOX2**	2.6%	4.91	2.4%	7.14
**KLF4**	0.2%	7.50	5.8%	0.00
**CTCF**	6.5%	10.82	5.2%	21.50
**POL2**	4.0%	12.04	1.5%	15.50
**TAF1**	4.5%	13.81	0.2%	3.33
**MYC**	0.9%	18.75	0.4%	0.00
**OCT4**	0.5%	19.00	2.0%	11.57
**BRG1**	7.9%	25.04	0.5%	7.00
**NANOG**	0.7%	61.00	2.6%	7.07
**HDAC1**	N/A	N/A	20.7%	9.24
**P300**	2.0%	18.67	10.1%	14.75
**HDAC2**	31.3%	6.65	31.8%	6.69
**SIN3**	19.3%	83.21	12.6%	258.00
	Histone Modifications			
**H3K9me3**	3.6%	0.57	5.8%	0.93
**H3K36me3**	5.0%	0.71	11.9%	1.26
**H2A.Z**	18.2%	2.23	3.3%	1.80
**H3K4me1**	33.2%	3.37	37.5%	2.82
**H3K27ac**	13.9%	3.39	14.8%	2.67
**H3K9ac**	15.4%	4.36	24.4%	3.25
**H3K27me3**	15.3%	4.61	14.2%	3.21
**H3K4me2**	26.2%	4.80	30.7%	4.70
**H3K4me3**	19.0%	5.85	22.2%	6.16

Table S6. Numbers and percentages of REST peaks in the three types.

	Number of peaks			Percent		
	**type I**	**type II**	**type III**	**type I**	**type II**	**type III**
**human all**	1,047	1,974	5,178	12.8%	24.1%	63.2%
**human conserved**	345	483	424	27.6%	38.6%	33.9%
**human non-conserved**	549	1,320	3,906	9.5%	22.9%	67.6%
**mouse all**	521	1,170	2,416	12.7%	28.5%	58.8%
**mouse conserved**	348	479	420	27.9%	38.4%	33.7%
**mouse non-conserved**	108	574	1,688	4.6%	24.2%	71.2%

Table S7. List of all mouse and human upregulated genes in REST depletion and the citations.

See separate Excel file.

Table S8. List of all disease associated genes and the data sources.

See separate Excel file.

Table S9.List of disease associated genes with a peak in both and human-specific REST peak.

Diseases/ Pathways	Peak in both species	Human specific peak
**AD**	ACTA1,APBA2,CACNA1D,CACNB4,CHRM2,CHRM4,CTNNA2,FZD4,KLC1,LEF1,MAPK10,MAPK11,MMP24,PCSK1,PCSK2,PRKCB,PRKCE,PRKCG,PRKCZ,PVRL1,TCF7L1,TRPC7,WNT2B	ACTG2,ADAM10,APBB1,APP,CACNA1C,CACNB2,CHRM1,CHRM3,DVL3,ERBB4,ERN2,GSK3B,LRP1B,LRP2,LRP5,LRP6,MAPK14,MAPK8,MAPKAPK2,MLLT4,MMP23B,NCSTN,NOTCH1,PCSK6,PKN3,PRKCA,PRKCD,PSEN2,TCF7L2,TRIM2,WNT3,WNT4,WNT7A,WNT8A
**ALS**	NEFH	ALS2,ERBB4,FUS,SETX,VAPB
**ASD**	ADCYAP1,AGAP1,AUTS2,CUX1,DCX,DLX5,GLRA1,GLRA2,GLRA3,GRM8,KCNN3,NRCAM,NRXN1,OMG,PRKCB,RELN,REST,SLC6A11	ADAM23,ADRA2A,CACNA1C,CADPS2,DDC,FYN,GABRB1,GABRG1,GABRR2,GRPR,ID2,LARGE,LRRN3,MBP,NCAM1,NLGN4X,PENK,PTEN,SCN2B,SLC1A6,SLC6A1,SYPL1,TSC1
**HD**	ACTA1,BDNF,DLG4,DNAH7,DYNLL2,GRIK2,GRIK3,GRIK4,GRIN1,GRIN2A,GRIN2C,GRIN3A,KALRN,MAPK10,MAP3K9,TAF4,TP73,TUBB3,VAT1L	ACTG2,AP2A2,ARL4C,ARPC5L,CAPN13,CREBBP,DNAH17,DNAH5,DNAH6,DNAH9,DYNC1I1,DYNC2H1,EP300,FOS,GRIN2B,GRIN2D,HTT,MAP3K11,MAPK8,RHOJ,TP63,TUBB2A,TUBB6
**HKG**	AP2S1,CALM1,ELAVL3,EXTL3,GABARAPL2,GRM4,ID3,KIFC3,LMO1,LTBP4,MAPK8IP1,PSMB2,RPL11,RPL17,RPL38,RPLP1,SRM,TACC1,YWHAQ	ADAM15,ARHGEF7,ATF4,CALM2,CENPB,CHIT1,CIZ1,CNTN1,COL6A1,COPE,COX7A2L,DAXX,EIF4A2,ENTPD6,FUS,GM2A,GNAI2,GP2,H2AFY,H6PD,HADHA,HLA-C,HPCAL1,HSBP1,JAK1,LDHA,LGALS9,MAP3K11,MAPKAPK2,MC2R,MIF,MYH9,NCL,NUDT3,NXF1,PFDN1,PGK1,PHGDH,PLXNB2,POLR2A,POLR2L,PSMA7,PSMB7,PTDSS1,PTMA,RPL10,RPL32,RPS14,RPS16,RPS2,SDC3,SLC25A3,SLC6A7,SPAG7,ST5,TAGLN,TALDO1,TCOF1,TIMM44,TKT,UQCRC1,ZFP36L1
**ID**	CTNND2	CLN8,IL1RAPL1,PDHX
**ND**	APLP1,BDNF,BSN,CDK5R2,ESR1,FGF14,NEFH,PRKCG,SLC1A1,SYP,WFS1	BCHE,BCL2,BCL2L1,CASP7,CDK5,CDK5R1,FGF2,GRB2,INSR,SGK1
**OS**	MAPK10,MAPK11	BCL2,DUSP4,DUSP6,DUSP7,MAP2K6,MAP3K4,MAPK14,MAPK8,MAPKAPK2,MAX,STAT1,STYX
**PD**	ATP1A3,DBH,FBXW11,MAPK10,MAPK11,PARK2,SRC,TOR2A,TPH2,TTBK1,YWHAQ	BTRC,FRK,FYN,MAPK14,MAPK8,PARK7,PLD2,PSMA7,PSMB7,SEPT4,SLC6A3,SNCAIP,TTBK2
**SZ**	ANKHD1,DAO,DRD3,NPAS3,NRG1,PPP3CA,RTN4R,SPTBN4,SYN2	APOL2,ATF4,CCDC141,DISC1,ERBB4,PDE4B,PDLIM5,PLXNA2,PPP1R1B,SYNE1,TNF
